# The Committee on Legislation and the Promotion of Homœopathy

**Published:** 1895-02

**Authors:** Edward F. Brady

**Affiliations:** Secretary Committee, 1315 Jefferson St., Kansas City, Mo.


### THE COMMITTEE ON LEGISLATION AND THE PROMOTION OF HOMŒOPATHY

(Created and appointed by the Missouri Institute of Homœopathy, Session 1894),

Whose address to the profession appeared in the November number of THE HOMŒOPATHIC PHYSICIAN at page 365, has published another circular from which the following extract is clipped:

Sinews of war. Money is essential and necessary in carrying forward the work we have in hand. It would not be just or equitable to lay this burden on the shoulders of an individual, or of a few. The Missouri Institute of Homœopathy generally, contributed what funds were in the treasury to start this movement, amounting to about $60. Now that we have put our hands to the plow, there will be no turning back; we must press forward to our goal. Committees will have to go to Jefferson City, and time and money must of necessity be spent, and the money must be raised by personal contributions. I ask all homœopaths interested in this work to contribute any sum which their interest in the cause of Homœopathy may suggest. Any sum will be thankfully received and applied toward the accomplishment of our aims and purposes. It would give me pleasure to write a personal letter to every homœopathic physician in the State, but the demands on my time in prosecuting this work makes it impossible, hence this circular letter to all. Come up to the measure of your patriotic duty, Doctor, and the end will be a victory. Fraternally yours,

EDWARD F. BRADY,
*Secretary Committee,*
1315 Jefferson St., Kansas City, Mo.

December 1st, 1894.

